# A Year's Work in the Medical Charities of London

**Published:** 1890-06-07

**Authors:** 


### A Year's Work in the Medical Charities of London.

[We were unable last week to give the following figures, embracing a correct and full account of the Income and Expenditure and the work done in 1889 by the Metropolitan Medical Charities. A summary of the whole will be found on page 144.]

### NEWINGTON AND SOUTH DISTRICT.

Comprising Battersea, Wandsworth, Tooting, Balham, Streatham, Brixton, Lambeth, Newington, Southwark, Bermondsey, Camberwell, Greenwich, Deptford, Lewisham, Blackheath, Woolwich, etc.

| No. of Beds. | No. of Beds Daily Occupied. | Hospitals. | In-patients. | Out-patients. | Total Expenditure. | Income. | | | Total Income. | Legacies over £100 not Included. |
|---|---|---|---|---|---|---|---|---|---|---|
| | | | | | | Charitable. | Proprietary. | Patients' Payments | | |
| | | | | | £ | £ | £ | £ | £ | £ |
| 600 | 418 | Guys' ... ... ... ... ... | 5,385 | 40,014 | 36,038 | 5,953 | 29,246 | 2,656 | 37,855 | ... |
| 24 | 20 | Miller... ... ... ... ... | 226 | 11,347 | 3,439 | 2,362 | 315 | ... | 2,677 | ... |
| 225 | 186 | Seamen's ... ... ... ... | 2,401 | 6,914 | 12,019 | 6,718 | 3,792 | 78 | 10,588 | 2,069 |
| 60 | 52 | Evelina, for Children ... ... | 603 | 5,520 | 5,378 | 3,789 | 723 | 57 | 4,569 | ... |
| 45 | 40 | Home for Sick Children ... ... | 277 | 1,649 | 1,452 | 1,232 | 3 | 312 | 1,547 | ... |
| 24 | 21 | General Lying-In ... ... ... | 484 | 1,091 | 2,971 | 714 | 2,258 | ... | 2,972 | ... |
| 50 | 46 | Royal, for Children and Women ... | 354 | 7,105 | 2,905 | 2,012 | 675 | 169 | 2,856 | ... |
| 11 | 9 | Gordon, for Fistula... ... ... | 158 | 505 | 960 | 599 | ... | 461 | 1,060 | ... |
| 14 | 10 | Royal South London Ophthalmic... | 113 | 6,323 | 981 | 752 | 90 | ... | 842 | ... |
| 10 | 2 | Hospital for Diseases of the Skin... | 17 | 5,873 | 959 | 335 | 93 | 619 | 1,047 | ... |
| 500 | 221 | Metropolitan Convalescent ... | 3,389 | ... | 6,361 | 5,546 | 411 | 57 | 6,014 | 1,956 |
| 1,563 | 1,025 | | 13,407 | 86,341 | 73,463 | 30,012 | 37,606 | 4,409 | 72,027 | 4,025 |
| | | Dispensaries. | | | | | | | | |
| ... | ... | Battersea, Provident ... ... | ... | 8,649 | 1,928 | 84 | 30 | 1,862 | 1,976 | ... |
| ... | ... | Brixton, &c. ... ... ... ... | ... | 5,857 | 550 | 584 | 57 | 80 | 721 | ... |
| ... | ... | Camberwell, Provident ... ... | ... | 9,673 | 1,817 | 536 | 83 | 1,298 | 1,917 | ... |
| ... | ... | Clapham ... ... ... ... ... | ... | 2,418 | 462 | 315 | ... | 107 | 422 | ... |
| ... | ... | Forest Hill ... ... ... ... | ... | 1,521 | 567 | 217 | ... | 339 | 556 | ... |
| ... | ... | Gipsy Hill ... ... ... ... | ... | 1,944 | 238 | 61 | ... | 173 | 234 | ... |
| ... | ... | Royal South London ... ... | ... | 6,330 | 710 | 635 | 108 | .. | 743 | ... |
| ... | ... | South Lambeth, &c. ... ... | ... | 2,682 | 635 | 383 | 6 | 227 | 616 | ... |
| ... | ... | South London Medical Aid ... | ... | 1,256 | 451 | 87 | 136 | 266 | 489 | ... |
| ... | ... | Wandsworth Common ... ... | ... | 1,145 | 168 | 50 | ... | 112 | 162 | ... |
| ... | ... | West Dulwich ... ... ... | ... | 444 | 45 | 43 | ... | 31 | 74 | ... |
| 1,563 | 1,025 | | 13,407 | 128,260 | 81,034 | 33,007 | 38,026 | 8,904 | 79,937 | 4,025 |

### CITY AND EAST CENTRAL DISTRICT.

Comprising the City, St. Luke's, Shoreditch, Finsbury, and Clerkenwell.

| No. of Beds. | No. of Beds Daily Occupied. | Hospitals. | In-patients. | Out-patients. | Total Expenditure. | Income. | | | Total Income. | Legacies over £100 not included. |
|---|---|---|---|---|---|---|---|---|---|---|
| | | | | | | Charitable. | Proprietary. | Patients' Payments. | | |
| | | | | | £ | £ | £ | £ | £ | £ |
| 76 | 47 | Metropolitan... ... ... ... | 489 | 9,482 | 5,813 | 3,390 | 318 | 740 | 4,448 | 261 |
| 160 | 133 | Royal Free ... ... ... ... ... | 2,019 | 24,374 | 10,111 | 3,974 | 1,109 | ... | 5,083 | 4,694 |
| 80 | 41 | Royal, for Diseases of the Chest ... | 407 | 5,609 | 4,987 | 3,145 | 263 | ... | 3,408 | 302 |
| 55 | 37 | North-Eastern for Children ... | 531 | 14,550 | 5,034 | 4,086 | 256 | 692 | 5,034 | 748 |
| 35 | 20 | City of London Lying-In ... ... | 422 | 1,499 | 3,977 | 367 | 2,841 | 21 | 3,229 | ... |
| 34 | 22 | St. Marks, for Fistula ... ... | 286 | 856 | 2,097 | 1,080 | 811 | ... | 1,891 | 2,334 |
| 100 | 96 | Royal London Ophthalmic ... | 2,337 | 26,865 | 6,495 | 2,733 | 998 | ... | 3,731 | 450 |
| 27 | 18 | City Orthopædic ... ... ... | 140 | 2,550 | 1,294 | 1,459 | 78 | ... | 1,537 | ... |
| 16 | 13 | Central London Throat and Ear ... | 230 | 6,257 | 2,122 | 787 | 29 | 1,465 | 2,281 | 520 |
| 583 | 427 | | 6,861 | 92,042 | 41,930 | 21,021 | 6,703 | 2,918 | 30,642 | 9,309 |
| | | Dispensaries. | | | | | | | | |
| ... | ... | City ... ... ... ... ... ... | ... | 5,007 | 1,375 | 1,344 | 184 | ... | 1,528 | ... |
| ... | ... | City of London and East London... | ... | 7,546 | 848 | 171 | 18 | 806 | 995 | ... |
| ... | ... | Farringdon General... ... ... | ... | 7,795 | 712 | 246 | ... | 380 | 626 | ... |
| ... | ... | Finsbury ... ... ... ... ... | ... | 18,000 | 907 | 467 | 122 | 318 | 907 | ... |
| ... | ... | Metropolitan ... ... ... | ... | 6,735 | 592 | 359 | 120 | 215 | 694 | ... |
| ... | ... | Royal General ... ... ... | ... | 3,838 | 832 | 1,048 | 243 | 97 | 1,388 | ... |
| ... | ... | Royal Maternity Charity ... ... | ... | 3,427 | 2,129 | 1,053 | 843 | ... | 1,896 | ... |
| 583 | 427 | | 6,861 | 144,390 | 49,325 | 25,709 | 8,233 | 4,734 | 38,676 | 9,309 |

### ST. MARYLEBONE AND WEST CENTRAL DISTRICT.

Comprising St. Marylebone, St. John's Wood, Bloomsbury, Holborn, etc.

| No. of Beds. | No. of Beds Daily Occupied. | Hospitals. | In-patients. | Out-patients. | Total Expenditure. | Income. Charitable. | Income. Proprietary. | Income. Patients' Payments. | Total Income. | Legacies over £100 not included. |
|---|---|---|---|---|---|---|---|---|---|---|
| | | | | | £ | £ | £ | £ | £ | £ |
| 36 | 28 | French ... ... ... ... ... | 366 | 3,862 | 2,520 | 2,856 | 317 | ... | 3,173 | ... |
| 20 | 13 | Italian ... ... ... ... ... | 232 | 2,723 | 1,037 | 2,061 | 45 | ... | 2,106 | ... |
| 51 | 47 | SS. John and Elizabeth ... ... | 96 | ... | 1,740 | 987 | 779 | ... | 1,766 | 450 |
| 307 | 254 | The Middlesex ... ... ... | 2,948 | 38,830 | 28,588 | 12,831 | 7,232 | ... | 20,063 | 35,376 |
| 81 | 81 | Alexandra for Children ... ... | 140 | 105 | 2,649 | 1,691 | 128 | 655 | 2,474 | ... |
| 30 | 29 | Home for Incurable Children ... | 37 | ... | 1,413 | 853 | 15 | 492 | 1,360 | ... |
| 181 | 91 | Hospital for Sick Children... ... | 928 | 18,896 | 11,300 | 6,942 | 1,108 | ... | 8,050 | 3,553 |
| 16 | 8 | British Lying-In ... ... ... | 148 | 600 | 1,499 | 522 | 1,004 | 4 | 1,530 | ... |
| 56 | 39 | Queen Charlotte's Lying-In ... | 995 | 1,179 | 3,482 | 2,521 | 310 | 13 | 2,844 | 521 |
| 26 | 22 | New Hospital for Women... ... | 265 | 4,068 | 2,172 | 1,375 | 140 | 568 | 2,083 | ... |
| 54 | 50 | Samaritan Free ... ... ... | 521 | 5,053 | 5,626 | 4,748 | 24 | ... | 4,772 | 200 |
| 175 | 148 | National for the Paralysed, &c. ... | 747 | 3,655 | 11,422 | 6,281 | 1,375 | 1,882 | 9,538 | 930 |
| 25 | 22 | Hospital for Epilepsy, &c.... ... | 94 | 751 | 2,221 | 1,530 | 19 | 909 | 2,458 | 300 |
| 10 | 9 | West End for Paralysis, &c. ... | 41 | 985 | 1,925 | 1,999 | ... | 534 | 2,533 | ... |
| 13 | 4 | Central London Ophthalmic ... | 155 | 8,014 | 1,045 | 734 | 54 | 306 | 1,094 | ... |
| 20 | 4 | Western Ophthalmic ... ... | 63 | 2,296 | 519 | 412 | 113 | 185 | 710 | ... |
| 35 | 31 | National Orthopædic ... ... | 141 | 714 | 1,179 | 603 | ... | 625 | 1,228 | ... |
| 4 | 2 | London Throat ... ... ... | 18 | 1,244 | 1,044 | 485 | 11 | 532 | 1,028 | ... |
| 94 | 55 | London Homœopathic ... ... | 711 | 9,486 | 4,634 | 2,515 | 2,285 | 392 | 5,192 | 15 |
| 25 | 12 | Establishment for Gentlewomen ... | 128 | ... | 2,291 | 795 | 89 | 1,058 | 1,942 | ... |
| 1,259 | 949 | | 8,774 | 102,461 | 88,306 | 52,741 | 15,048 | 8,155 | 75,944 | 41,480 |
| | | Dispensaries. | | | | | | | | |
| ... | ... | Margaret Street Infirmary ... | ... | 2,019 | 484 | 352 | 148 | ... | 500 | ... |
| ... | ... | Portland Town ... ... ... | ... | 1,453 | 177 | 293 | ... | 10 | 303 | ... |
| ... | ... | Portobello Road ... ... ... | ... | 248 | 76 | 17 | 4 | 57 | 78 | ... |
| ... | ... | St. John's Wood Provident ... | ... | 4,130 | 521 | 242 | 18 | 251 | 511 | ... |
| ... | ... | St. Marylebone General ... ... | ... | 4,330 | 786 | 391 | 131 | 280 | 802 | ... |
| ... | ... | Western General ... ... ... | ... | 19,828 | 1,369 | 1,172 | 42 | ... | 1,214 | 200 |
| 1,259 | 949 | | 8,774 | 134,469 | 91,719 | 55,208 | 15,391 | 8,753 | 79,352 | 41,680 |

### KENSINGTON AND WEST DISTRICT.

Comprising Kensington, Paddington, Bayswater, Kilburn, Chelsea, Brompton, Fulham, Hammersmith, Chiswick, Brentford, Acton, Ealing, etc.

| No. of Beds. | No. of Beds Daily Occupied. | Hospitals. | In-patients. | Out-patients. | Total Expenditure. | Income. Charitable. | Income. Proprietary. | Income. Patients' Payments. | Total Income. | Legacies over £100 not included. |
|---|---|---|---|---|---|---|---|---|---|---|
| | | | | | £ | £ | £ | £ | £ | £ |
| 351 | 316 | St. George's ... ... ... ... ... | 4,029 | 26,459 | 27,383 | 10,765 | 13,293 | ... | 24,058 | 21,315 |
| 281 | 251 | St. Mary's ... ... ... ... ... | 3,451 | 27,265 | 23,275 | 11,938 | 2,668 | ... | 14,606 | 11,389 |
| 10i | 87 | West London ... ... ... ... | 1,369 | 21,980 | 6,098 | 4,802 | 71 | ... | 4,873 | ... |
| 321 | 275 | Hospital for Consumption... ... | 1,669 | 13,845 | 25,377 | 15,694 | 7,414 | ... | 23,108 | 4,545 |
| 23 | 17 | Belgrave, for Children ... ... | 220 | 2,555 | 1,526 | 1,316 | 31 | ... | 1,347 | 140 |
| 24 | 24 | Cheyne, for Sick & Incurable Children | 35 | ... | 1,458 | 1,594 | 85 | 238 | 1,917 | ... |
| 27 | 23 | Paddington Green, for Children ... | 400 | 10,789 | 2,338 | 2,586 | 146 | 154 | 2,886 | 717 |
| 90 | 79 | Victoria, for Children ... ... | 1,193 | 14,427 | 5,880 | 3,982 | 921 | 309 | 5,212 | ... |
| 60 | 39 | Chelsea, for Women ... ... | 404 | 2,504 | 3,882 | 2,473 | 45 | 588 | 3,106 | ... |
| 105 | 80 | Cancer ... ... ... ... | 679 | 1,099 | 8,376 | 5,666 | 1,330 | ... | 6,996 | 7,573 |
| 135 | 96 | Female Lock... ... ... ... | 721 | ... | 4,018 | 3,064 | ... | 744 | 3,808 | 475 |
| 20 | 14 | Male Lock ... ... ... ... | 251 | 3,434 | 1,563 | 192 | ... | 1,165 | 1,357 | ... |
| 31 | 30 | St. Mary Magdalene's Home ... | 59 | 17 | 839 | 413 | 10 | 391 | 814 | ... |
| 1,569 | 1,331 | | 14,480 | 124,374 | 112,013 | 64,485 | 26,014 | 3,589 | 94,088 | 46,154 |
| | | Dispensaries. | | | | | | | | |
| ... | ... | Brompton Provident ... ... | ... | 944 | 450 | 156 | 7 | 279 | 442 | ... |
| ... | ... | Chelsea, &c. ... ... ... | ... | 4,759 | 585 | 358 | 166 | ... | 524 | 450 |
| ... | ... | Kensal Town, Provident ... ... | ... | 693 | 344 | 70 | ... | 275 | 345 | ... |
| ... | ... | Kensington ... ... ... ... | ... | 4,825 | 662 | 724 | 39 | ... | 763 | ... |
| ... | ... | Kilburn, Maida Vale ... ... | ... | 2,152 | 442 | 578 | 40 | ... | 618 | 200 |
| ... | ... | Kilburn Provident ... ... ... | ... | 3,669 | 1,047 | 97 | 83 | 866 | 1,046 | ... |
| ... | ... | Notting Hill Provident ... ... | ... | 874 | 277 | 164 | 24 | 88 | 276 | ... |
| ... | ... | Paddington Provident ... ... | ... | 7,046 | 584 | 198 | 26 | 380 | 604 | ... |
| ... | ... | Royal Pimlico Provident ... ... | ... | 5,846 | 941 | 320 | 16 | 566 | 902 | ... |
| ... | ... | SS. Paul and Barnabas ... ... | ... | 2,694 | 506 | 361 | 4 | 135 | 500 | ... |
| ... | ... | Westbourne Provident ... ... | ... | 3,378 | 412 | 81 | 15 | 273 | 369 | ... |
| ... | ... | Pimlico Provident ... ... ... | ... | 2,141 | 425 | 5 | ... | 304 | 309 | ... |
| 1,569 | 1,331 | | 14,480 | 163,395 | 118,688 | 67,597 | 26,434 | 6,755 | 100,786 | 46,804 |

### ISLINGTON AND NORTH-WEST DISTRICT.

Comprising Islington, Holloway, Highbury, Hampstead, Highgate, St. Pancras, Stoke Newington, Tottenham, etc.

| No. of Beds. | No. of Beds Daily Occupied. | Hospitals. | In-patients. | Out-patients. | Total Expenditure. | Income. Charitable. | Income. Proprietary. | Income. Patients' Payments. | Total Income. | Legacies over £100 not included. |
|---|---|---|---|---|---|---|---|---|---|---|
| | | | | | £ | £ | £ | £ | £ | £ |
| 68 | 61 | Great Northern Central ... ... | 861 | 15,563 | 6,166 | 3,835 | 493 | 385 | 4,713 | 584 |
| 47 | 39 | North-West London ... ... | 537 | 13,906 | 2,800 | 2,219 | 82 | 77 | 2,378 | ... |
| 103 | 59 | Tottenham ... ... ... ... | 801 | 7,449 | 4,391 | 2,372 | 736 | 577 | 3,685 | 711 |
| 207 | 179 | University College ... ... ... | 3,007 | 41,273 | 18,914 | 7,620 | 5,152 | 115 | 12,887 | 14,356 |
| 48 | 45 | North London Consumption ... | 348 | 2,898 | 4,823 | 4,816 | 82 | 16 | 4,914 | ... |
| 220 | 54 | London Fever ... ... ... | 538 | ... | 7,293 | 6,499 | 1,681 | 1,966 | 10,146 | 203 |
| 77 | 57 | London Temperance ... ... | 735 | 2,873 | 5,516 | 4,487 | 377 | 245 | 5,109 | 350 |
| 18 | 10 | Hampstead Home Hospital ... | 118 | ... | 1,837 | 386 | 554 | 615 | 1,555 | ... |
| 28 | 23 | Invalid Asylum ... ... ... | 207 | ... | 1,114 | 639 | 92 | 275 | 1,006 | 800 |
| 816 | 527 | | 7,152 | 83,962 | 5,284 | 32,873 | 9,249 | 4,271 | 46,393 | 17,004 |
| | | **Dispensaries.** | | | | | | | | |
| ... | ... | Camden Provident ... ... ... | ... | 1,092 | 314 | 25 | ... | 295 | 320 | ... |
| ... | ... | Child's Hill ... ... ... ... | ... | 1,128 | 238 | 43 | 9 | 191 | 243 | ... |
| ... | ... | Hampstead Provident ... ... | ... | 10,158 | 889 | 300 | 9 | 588 | 897 | ... |
| ... | ... | Holloway and North Islington ... | ... | 4,278 | 829 | 502 | 26 | 338 | 866 | 761 |
| ... | ... | Islington ... ... ... ... | ... | 14,006 | 874 | 282 | 53 | 582 | 917 | ... |
| ... | ... | St. Pancras and Northern... ... | ... | 2,603 | 564 | 329 | 106 | 91 | 526 | ... |
| ... | ... | Stamford Hill, &c. ... ... | ... | 4,075 | 546 | 497 | 141 | ... | 638 | ... |
| 816 | 527 | | 7,152 | 121,302 | 57,108 | 34,851 | 9,593 | 6,356 | 50,800 | 17,765 |

### WESTMINSTER DISTRICT.

Comprising Westminster City and Liberties.

| No. of Beds. | No. of Beds Daily Occupied. | Hospitals. | In-patients. | Out-patients. | Total Expenditure. | Income. Charitable. | Income. Proprietary. | Income. Patients' Payments. | Total Income. | Legacies over £100 not included. |
|---|---|---|---|---|---|---|---|---|---|---|
| | | | | | £ | £ | £ | £ | £ | £ |
| 175 | 143 | Charing Cross ... ... ... ... | 1,948 | 21,432 | 15,324 | 5,635 | 3,313 | ... | 8,948 | ... |
| 218 | 163 | King's College ... ... ... | 2,189 | 19,592 | 16,942 | 11,267 | 1,744 | 76 | 13,087 | 7,408 |
| 205 | 175 | Westminster... ... ... ... | 2,572 | 25,031 | 13,278 | 5,551 | 2,713 | ... | 8,264 | 1,026 |
| 132 | 111 | Ventnor, for Consumption | 759 | ... | 11,244 | 4,287 | 1,761 | 2,936 | 8,984 | 3,750 |
| 14 | 11 | Grosvenor, for Women & Children | 103 | 2,232 | 1,115 | 586 | 16 | 472 | 1,074 | ... |
| 66 | 49 | Hospital for Women ... | 632 | 4,896 | 6,534 | 3,410 | 59 | 1,249 | 4,718 | 2,450 |
| 25 | 18 | National, for Diseases of Heart, &c. | 114 | 2,168 | 1,949 | 1,626 | 3 | 358 | 1,987 | ... |
| 50 | 25 | Royal Westminster Ophthalmic ... | 323 | 7,956 | 2,067 | 2,252 | 332 | 35 | 2,619 | 400 |
| 50 | 50 | Royal Orthopædic ... ... ... | 129 | 1,176 | 1,923 | 1,326 | 374 | 84 | 1,784 | 304 |
| 20 | 18 | Hospital for Diseases of the Throat | 419 | 6,593 | 2,935 | 2,291 | 100 | 2,558 | 4,949 | 675 |
| 9 | 4 | Royal Ear ... ... ... ... | 102 | 2,765 | 698 | 160 | 17 | 582 | 759 | ... |
| ... | ... | Dental ... ... ... ... | ... | 34,400 | 1,821 | 1,730 | 180 | 275 | 2,185 | ... |
| 964 | 767 | | 9,290 | 128,241 | 75,830 | 40,121 | 10,612 | 8,625 | 59,358 | 16,013 |
| | | **Dispensaries.** | | | | | | | | |
| ... | ... | London Medical Mission ... ... | ... | 3,781 | 1,051 | 947 | 13 | ... | 960 | ... |
| ... | ... | Public... ... ... ... ... | ... | 3,654 | 708 | 417 | 178 | ... | 595 | ... |
| ... | ... | St. George and St. James ... ... | ... | 3,415 | 548 | 377 | 20 | 31 | 428 | ... |
| ... | ... | St. George, Hanover Square | ... | 1,727 | 700 | 453 | 20 | 143 | 616 | ... |
| ... | ... | Western ... ... ... ... | ... | 4,034 | 1,011 | 428 | 384 | 289 | 1,101 | 375 |
| ... | ... | Westminster General ... ... | ... | 5,150 | 592 | 329 | 152 | 55 | 536 | ... |
| 964 | 767 | | 9,290 | 150,002 | 80,440 | 43,072 | 11,379 | 9,143 | 63,594 | 16,388 |

### STRATFORD AND EAST-END DISTRICT.

Comprising Bethnal Green, Tower Hamlets, West Ham, Whitechapel, Hackney, Stepney, Limehouse, Poplar, and the East.

| No. of Beds. | No. of Beds Daily Occupied. | Hospitals. | In-patients. | Out-patients. | Total Expenditure. | Income. Charitable. | Income. Proprietary. | Income. Patients' Payments. | Total Income. | Legacies over £100 not included. |
|---|---|---|---|---|---|---|---|---|---|---|
| | | | | | £ | £ | £ | £ | £ | £ |
| 125 | 105 | German ... ... ... ... | 1,199 | 23,326 | 9,388 | 6,626 | 2,362 | 364 | 9,352 | 633 |
| 776 | 622 | London ... ... ... ... | 8,503 | 109,839 | 59,423 | 21,957 | 27,640 | 219 | 49,816 | 25,490 |
| 50 | 37 | Poplar... ... ... ... ... | 745 | 12,057 | 3,101 | 3,156 | 415 | ... | 3,571 | ... |
| 164 | 109 | City of London for Dis. of the Chest | 962 | 14,847 | 10,298 | 8,295 | 479 | ... | 8,774 | 939 |
| 102 | 78 | East London for Children ... ... | 1,074 | 18,218 | 6,557 | 7,110 | 301 | ... | 7,411 | 261 |
| 92 | 53 | Mrs. Gladstone's Home ... ... | 915 | ... | 1,251 | 852 | 372 | ... | 1,224 | ... |
| 1,309 | 1,004 | | 13,398 | 178,287 | 90,018 | 47,996 | 31,569 | 583 | 80,148 | 27,323 |
| | | **Dispensaries.** | | | | | | | | |
| ... | ... | Eastern ... ... ... ... | ... | 6,421 | 690 | 297 | 284 | 59 | 640 | ... |
| ... | ... | Hackney Provident... ... ... | ... | 1,379 | 157 | ... | ... | 213 | 213 | ... |
| ... | ... | London ... ... ... ... | ... | 2,839 | 425 | 128 | 248 | ... | 376 | ... |
| ... | ... | Queen Adelaide's ... ... ... | ... | 4,856 | 435 | 589 | 156 | ... | 745 | ... |
| ... | ... | Tower Hamlets ... ... ... | ... | 3,894 | 594 | 323 | 21 | 124 | 468 | ... |
| ... | ... | West Ham, etc. ... ... ... | ... | 17,997 | 850 | 848 | 176 | ... | 1,024 | ... |
| 1,309 | 1,004 | | 13,398 | 215,673 | 93,169 | 50,181 | 32,454 | 979 | 83,614 | 27,323 |

### SUMMARY.

It will be seen from the following summary that the Voluntary Hospitals and Medical Charities of London, during the 12 months ending 31st December, 1889, relieved over one million, one hundred, and thirty thousand patients, at a cost of £571,483, against an Ordinary Income of only £496,759.

| No. of Beds. | No. of Beds Daily Occupied. | Hospitals and Dispensaries. | In-patients. | Out-patients. | Total Expenditure. | Income. | | | Ordinary Income. | Legacies over £100 not included. |
|---|---|---|---|---|---|---|---|---|---|---|
| | | | | | | Charitable. | Proprietary. | Patients' Payments. | | |
| | | | | | £ | £ | £ | £ | £ | £ |
| 1,563 | 1,025 | Newington and South District... | 13,407 | 128,260 | 81,034 | 33,007 | 38,026 | 8,904 | 79,937 | 4,025 |
| 583 | 427 | City and East Central District... | 6,861 | 144,390 | 49,325 | 25,709 | 8,233 | 4,734 | 38,676 | 9,309 |
| 964 | 767 | Westminster District ... ... | 9,290 | 150,002 | 80,440 | 43,072 | 11,379 | 9,143 | 63,594 | 16,388 |
| 1,259 | 949 | St. Marylebone and West Central District ... ... ... | 8,774 | 134,469 | 91,719 | 55,208 | 15,391 | 8,753 | 79,352 | 41,680 |
| 1,569 | 1,331 | Kensington and West District... | 14,480 | 163,395 | 118,688 | 67,597 | 26,434 | 6,755 | 100,786 | 46,804 |
| 816 | 527 | Islington and North-West District | 7,152 | 121,302 | 57,108 | 34,851 | 9,593 | 6,356 | 50,800 | 17,765 |
| 1,309 | 1,004 | Stratford and East-End District | 13,398 | 215,673 | 93,169 | 50,181 | 32,454 | 979 | 83,614 | 27,323 |
| 8,063 | 6,030 | | 73,362 | 1,057,491 | 571,483 | 309,625 | 141,510 | 45,624 | 496,759 | 163,294 |

### SCRAPS AND GLEANINGS.

Colonel North has given £500 to the French Hospital.

In Buffalo there is a club of bicyclists, all the members of which are doctors.

M. Carnot visits all the chief hospitals in the towns he stays at during his present tour.

Over £1,000 was subscribed for the Evelina Hospital at the late dinner in aid of that charity.

Mrs. Harper has died in New York under chloroform, administered for the extraction of a tooth.

The Duke of Connaught opened the Jubilee Hospital at Victoria, British Columbia, on May 21st.

Cambridge proposes to bestow honorary degrees on Sir Andrew Clark and Mr. Jonathan Hutchinson.

Mrs. Goslett has sent out a water bed to Sister Francis, matron of St. Luke's Hospital, Vancouver.

At the anniversary festival of the Royal Masonic Institution for Girls, the subscriptions realised £11,000.

At the dinner in aid of the North London Consumption Hospital, the subscriptions amounted to £4,000.

The Samaritan Free Hospital for Women cleared over £1,000 by the bazaar lately held in the Portman Rooms.

The Infectious Hospital for Epsom District will probably cost £400 a-year, and be built close to Banstead station.

The annual Beard concert by the pupils of the School for the Indigent Blind was held on May 22nd, and well attended.

Dr. Chaffey and Mr. G. Morgan have been elected to the medical staff of the Alexandra Hospital for Children, Brighton.

Sir Andrew Clark has lately delivered a public address in which he avowed his deep belief in the truths of Christianity.

St. John's Hospital for Diseases of the Skin has been registered as a benevolent society under the Friendly Societies Act.

A Mrs. Darby, residing at Deal, has just entered on her 102nd year. She is in good health and in the enjoyment of all her faculties.

The Pharmaceutical Society held its annual dinner on May 21. Mr. J. Hutchinson responded to the toast of "The Medical Profession."

Dr. John Abercrombie, F.R.C.P., has been elected physician to the Charing Cross Hospital in the place of Dr. A. Julius Pollock, deceased.

A young man named Panting has been poisoned at Birmingham by eating chocolate cream containing copper, probably derived from a dirty mould.

A leper mendicant at Bombay, who was found ill in the street, was refused admission to the hospital. The man afterwards died in a public conveyance.

The muzzling order in some counties has been amended for an order commanding that every dog wear a collar bearing the name and address of the owner.

A dinner was given to Surgeon Parke, on June 6th, at the Criterion by his professional brethren. The list of stewards included names of all the leading doctors.

It is to be hoped the Eton Mission will secure the Hackney Marshes as a recreation ground. They have Sir Charles Russell on their side, which looks like victory.

Twelve Alpine soldiers of the 3rd Regiment are in hospital in Turin, suffering from ophthalmia and frost-bites contracted during a march of fifteen hours in the snow.

Dr. Symes Thompson presided at the twelfth annual meeting of the Ealing College for the Deaf, when an interesting exhibition of the method of tuition was given.

The Society of Mental Medicine are taking measures to obtain the erection in Paris of a special hospital and *clinique*, in order to provide means of studying mental diseases.

Earl Brownlow presided over the thirty-ninth annual meeting of the Royal Cambridge Asylum, when seven widows out of fifty approved candidates were elected for admission.

Upwards of 10,500 dogs have been received at the Dogs' Home, Battersea, since January 1, 1890, and the work at the Home now renders it necessary to increase the staff of men.

Queen Isabella, of Spain, visited the Hospital of St. John and St. Elizabeth, Great Ormond Street, and was so pleased with all she saw, she left a handsome donation behind her.

The Rowland Hill Benevolent Fund for the widows of the Post office employés has benefitted to the extent of nearly £240 by the happy thought of issuing the special Jubilee postcards.

The French operations in Dahomey have been temporarily suspended. The crews of the French ships off the coast are suffering severely from fever. Over seventy men out of a total of 350 are on the sick list.

Owing to a reported outbreak of cholera at Bagdad and Mosul, Dr. Elisseneff has left St. Petersburg on an official mission of sanitary inspection on the frontiers of Asia Minor. He will also visit Persia.

A new park, which has been presented to the inhabitants of Morley by the Earl of Dartmouth, was opened on May 24th by his lordship, who was accompanied by the countess. A drinking fountain, erected in commemoration of the Queen's Jubilee, was also unveiled.

Considerable havoc is being caused among farmers' corn in West Suffolk by a plague of rats. In the village of Lidgate on four stacks of wheat being threshed out over a thousand rats were killed as well as a great number of mice. The rats are in some cases as remarkable for their size as for their numbers.

### THE STUDENTS' COLUMN.

### UNIVERSITY OF LONDON.

M.B. EXAMINATION, MAY, 1890.

The following is the pass list:—

First Division.—Earle, E. R. C., University College; Evans, H. M., University College; Gill, J. McD., Guy's Hospital; Horden, H. H., University College; Jones, S. H., St. Thomas's Hospital; Lord, E. E., B.Sc., Owens College and Manchester Royal Infirmary; Pasmore, E. S., University College; Reichardt, E. N., St. Bartholomew's Hospital.

Second Division.—Bannister, M., Owens College and Manchester Royal Infirmary; Blackler, H. J., Guy's Hospital; Bryett, L. T. F., King's College; Bueno de Mesquita, S., Guy's Hospital; Davis, A. H., St. Bartholomew's Hospital; Frames, A. C., St. Bartholomew's Hospital; Hill, L. E., University College; Hinds, T. W., University College; McLaren, K., St. Bartholomew's Hospital; Sadler, E. A., Queen's College, Birmingham; Shaw, G., Westminster Hospital.

### THE HOMER BIRTHDAY BOOK.

It is not a little strange that of all the sources from which birthday books have been derived, it was not until the present year that it has occurred to any enthusiastic scholar to avail himself of the beauties of Homer. The blank thus left has now been filled by a charming little book of selections from the "Iliad" and the "Odyssey." It is beautifully got up, and is dedicated by permission to, and acknowledged by, Mr. Gladstone. We have not the pleasure of knowing the compiler's name, who modestly signs "V. E. G." He—or is it she?—may be warmly complimented on the success of the new departure.

